# Enhanced Optical and Electronic Properties of Silicon Nanosheets by Phosphorus Doping Passivation

**DOI:** 10.3390/ma16031079

**Published:** 2023-01-26

**Authors:** Ye Lei, Deren Yang, Dongsheng Li

**Affiliations:** State Key Laboratory of Silicon Materials and School of Materials Science and Engineering, Zhejiang University, Hangzhou 310027, China

**Keywords:** silicon nanosheet, phosphorus doping, photoelectric properties

## Abstract

In this paper, we use the spin-on-dopant technique for phosphorus doping to improve the photoelectric properties of soft-chemical-prepared silicon nanosheets. It was found that the luminescence intensity and luminescence lifetime of the doped samples was approximately 4 fold that of the undoped samples, due to passivation of the surface defects by phosphorus doping. Meanwhile, phosphorus doping combined with high-temperature heat treatment can reduce the resistivity of multilayer silicon nanosheets by 6 fold compared with that of as-prepared samples. In conclusion, our work brings soft-chemical-prepared silicon nanosheets one step closer to practical application in the field of optoelectronics.

## 1. Introduction

Since the discovery of graphene in 2004, two-dimensional (2D) nanomaterials have received more attention, including single-element 2D materials such as graphene and phosphorene and metal dichalcogenides represented by MoS_2_ [1,2,3,4]. The special 2D structure with the quantum confinement effect provide abnormal photonic, magnetic, catalytic, and electronic properties, conferring them with outstanding behaviors in sensors, ferroelectricity, catalysis, field-effect transistors, batteries, supercapacitors, and thermoelectricity. As the group ⅣA element, two-dimensional silicon (silicon nanosheet) is expected to have superior properties similar to graphene. Furthermore, it also preserves the advantages of silicon material, which is the most important material in modern life. Therefore, two-dimensional silicon may have broad application prospects in the fields of electricity, optics, chemistry and even biology [5,6,7,8,9,10].

At present, the main preparation methods of silicon nanosheets are chemical vapor deposition (CVD), molecular beam epitaxy (MBE) and the soft chemical method. The CVD and MBE methods are in the “bottom-up” growth mode, which can accurately regulate the thickness of silicon nanosheets by controlling the growth time or the growth rate, resulting in high-quality silicon nanosheet crystals [11,12]. Because these two methods adopt a high-vacuum or hydrogen atmosphere as the growth environment, the surface of the prepared silicon nanosheets are mainly composed of dangling bonds and Si-H bonds. However, the need for a large flux of hydrogen as a carrier gas, the low growth efficiency, expensive growth equipment and other deficiencies make these two methods not suitable for practical production. In contrast, the soft chemical method has the advantages of a simple process and easy mass production. The soft chemical method is a “top-down” preparation method—the chemical reaction will inevitably damage the crystal structure of the original material, so the silicon nanosheets obtained by this method have poorer crystal quality compared with the former. In order to weaken the interaction between the silicon layers in the original material and enhance their stability in solution or air, silicon nanosheets prepared by the soft chemical method usually require the introduction of surface modifiers through connecting a large number of organic groups on the surface [13,14].

Based on the two-dimensional crystal structure with low defect density, the silicon nanosheets prepared by the CVD and MBE methods show excellent photoelectric performance. The silicon nanosheets prepared by the CVD method have achieved tunable luminescence in UV–VIS band, and on this basis, white light organic light-emitting diode (OLED) was obtained [15]. Single-layer silicon nanosheets prepared by the MBE method have a graphene-like band structure and exhibit bipolar electrical characteristics under the regulation of gate voltage, with a resistivity of approximately 2.04 × 10^−3^ Ω · cm and a carrier mobility of approximately 100 cm^2^ V^−1^s^−1^ at room temperature [16]. Silicon nanosheets prepared by the soft chemical method also have a quantum confined effect and photoluminescence [17,18], but the uncontrollability of the thickness and the adverse effect of the crystal defects on the luminescence property limit the application of silicon nanosheets prepared by the soft chemical method in light-emitting devices. In addition, the surface defects of silicon nanosheets prepared by the soft chemical method capture the carriers and the surface modifiers impede the transmission of electrons between the nanosheets, resulting in poor conductivity, with an average resistivity of 7.24 Ω · cm and a carrier mobility of 36 cm^2^ V^−1^s^−1^ [19,20].

Referring to the relevant studies on doping of silicon nanocrystals, we found that phosphorus doping can passivate the dangling bonds on the surface of silicon nanomaterials, thus reducing the non-radiative recombination channels and carrier traps [21,22,23]. Therefore, we believe that phosphorus doping for silicon nanosheets fabricated by the soft chemical method will pave a way to improve their photoelectric properties. In this paper, the effects of phosphorus doping on photoelectric properties of silicon nanosheets was investigated. It is proved that phosphorus doping can effectively overcome the problem of high defect density on the surface of soft-chemical-prepared silicon nanosheets, and significantly improve the luminescence intensity, luminescence life and electrical conductivity of the material, which is superior to traditional surface modification method using organic modifiers.

## 2. Materials and Methods

We selected Zintel compound CaSi_2_ (Ca ≈ 30%, Alfa Aesar, Haverhill, MA, USA) composed of alternating layers of calcium ions and silicon ions as the original reactant and CuCl_2_ (98%, Shanghai Macklin Biochemical Co., Ltd., Shanghai, China) as the oxidant to produce silicon nanosheets by the de-intercalation reaction under the condition of water bath heating at 40 °C. CuCl_2_ oxidizes the negative silicon ions in CaSi_2_, breaking the ionic bonds within the compound, allowing the calcium ions between the silicon layers to desorbed and enter the solvent, leaving only the layered silicon skeleton. Then, ethanol is added for ultrasonic treatment, and the silicon particles with weak interaction between layers are separated into ultra-thin silicon nanosheets by mechanical force. Finally, the remaining reactants or insufficiently stripped silicon particles in the samples were removed by low-speed centrifugation, and the supernatant which contained ultra-thin silicon nanosheets was collected for subsequent experiments.

Phosphorus doping is realized by the spin-on-dopant method. First, the silicon nanosheets stored in anhydrous ethanol were coated on SiO_2_/Si (the thickness of SiO_2_ is 300 nm) substrate and dried at 60 °C, and liquid phosphorus dopant (P509, Filmtronics, Butler, PA, USA) was coated on the silicon substrate and dried at 200 °C for approximately 30 min. Then, the two substrates placed opposite each other with an interval of 400 µm were heated in the annealing furnace under vacuum environment to 925–1025 °C for 5–10 min. Finally, the cooled doped samples were rinsed quickly in 1% hydrofluoric acid solution to remove the surface oxide layer and residual doping sources. The undoped samples used as references underwent the same heat treatment process on SiO_2_/Si substrate.

## 3. Results and Discussion

**Figure 1:**
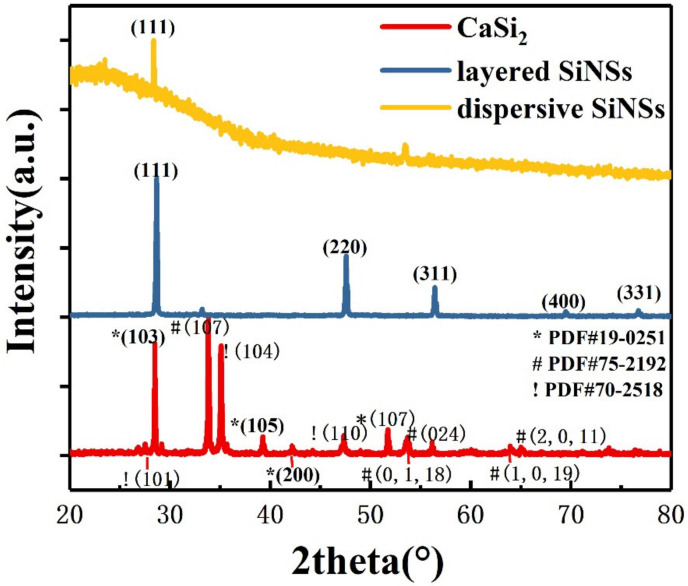
X-ray diffraction (XRD) spectrum of the original reactant CaSi_2_ (red line), as-prepared layered silicon nanosheets (blue line) and dispersive silicon nanosheets (yellow line).

Figure 1 shows the X-ray diffraction (XRD) patterns of the original reactant CaSi_2_, layered silicon nanosheets after CuCl_2_ oxidization and dispersive silicon nanosheets after ultrasonic treatment. The CaSi_2_ (red line) we used mixed three crystalline phases with PDF numbers shown in Figure 1. The diffraction peak at approximately 28° demonstrates that each layer of Si atoms in CaSi_2_ is arranged in the same way as the (111) crystal plane in crystalline silicon.

As can be seen from the XRD pattern of the layered silicon nanosheets (blue line), the reaction product corresponds to the cubic-phase crystalline silicon. Though there is a diffraction peak of a residual byproduct CuCl, no sign of any original reactant CaSi_2_ (red line) is found in the pattern. It is proved that Ca^2+^ can be effectively removed through the redox reaction of CaSi_2_ and CuCl_2_, and the silicon skeleton in CaSi_2_ can be preserved.

Only one diffraction peak can be observed in the XRD pattern of dispersive silicon nanosheets (yellow line in Figure 1). It indicates that they no longer have the periodicity of crystalline silicon in other directions, reflecting the quasi two-dimensional characteristics. The diffraction peak corresponds to the (111) crystal plane, illustrating that the procedure is inserting copper particles between adjacent silicon layers of CaSi_2_ to weaken the interaction, making them easy to peel off in the perpendicular direction under the external force. Compared with the layered silicon nanosheets, partial amorphous will occur in the dispersive ones, resulting in the intensity of the diffraction peak weakening.

**Figure 2:**
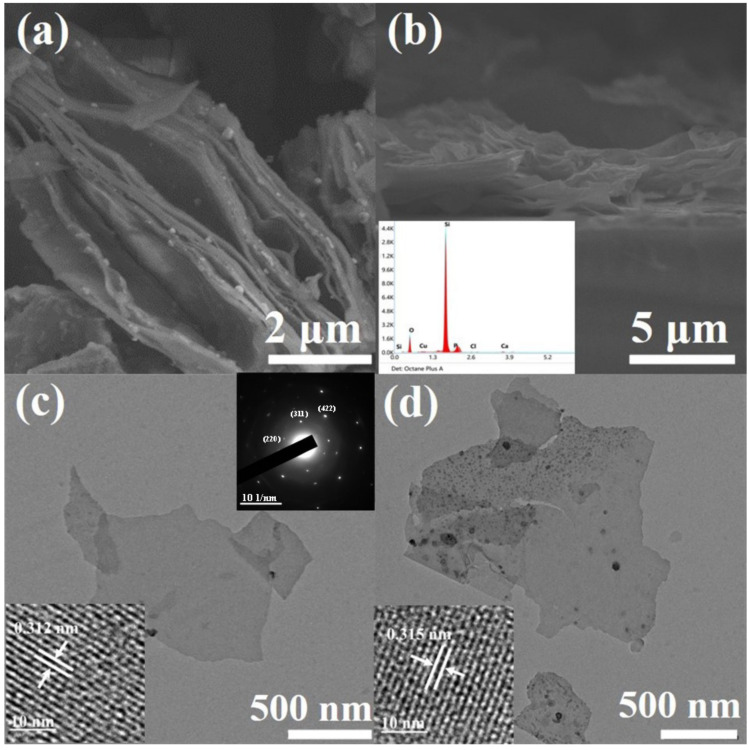
Scanning electron microscope (SEM) and transmission electron microscope (TEM) images of silicon nanosheets. (**a**) Top-view image of undoped layered silicon nanosheets; (**b**) cross-section image of doped layered silicon nanosheets with energy dispersive spectroscopy (EDS) as inset; (**c**) TEM image of undoped dispersive silicon nanosheets with high resolution transmission electron microscope (HRTEM) image and selected area electron diffraction (SAED) pattern as insets; (**d**) TEM image of doped dispersive silicon nanosheets with HRTEM image as inset.

Figure 2a shows the scanning electron microscope (SEM) image of layered silicon nanosheets obtained by a CaSi_2_ de-intercalation reaction. It can be seen from the image that there are many interspaces between layers, which are formed by the insertion and desorption of Cu particles [24]. As we know, due to thermodynamic instability, when the lamella are very thin, they tend to form folds and undulations to release energy. Therefore, the silicon nanosheets present as wavy lamella as shown in Figure 2a.

By comparing the SEM images of the undoped (Figure 2a) and doped samples (Figure 2b), it can be seen that there is no obvious difference in morphology between them. The doped nanosheets can still maintain discrete without re-bonding during the heat treatment process. A small peak of P element can be obviously observed in the energy dispersive spectroscopy (EDS) spectrum (inset of Figure 2b) of doped samples, and the weight percentage of P element is roughly 4.5%.

Transmission electron microscope (TEM) characterization of the silicon nanosheets obtained by ultrasonic peeling showed that the samples were uniform in thickness and smooth in surface. As can be seen from Figure 2c,d, the size of nanosheets varies, with side lengths ranging from 1 to 8 µm. By contrast with the broken particles in the image, the thickness of the silicon nanosheet can be roughly determined to be less than 10 nm. The typical diffraction spots of cubic silicon can be found in the indexed selected area electron diffraction (SAED) pattern of multilayer silicon nanosheets (inset at upper-right corner of Figure 2c), which can be corresponding to the XRD peaks of layered silicon nanosheets (blue line in Figure 1). High resolution transmission electron microscope (HRTEM) image (inset at the left bottom of Figure 2c) prove that these ultra-thin silicon nanosheets still retain clear lattice streaks with a lattice spacing of 0.312 nm, corresponding to the (111) crystal plane of cubic silicon, which is consistent with the XRD diffraction peak of dispersive silicon nanosheets (yellow line in Figure 1). Similar to XRD results, some amorphous regions, especially the edges, appear in the dispersed silicon nanosheets. This is because nanosheets less than 10 nm thick are sensitive to oxygen in the air, deionized water and oxygen free radicals generated by high-speed electron beams, and easily oxidized to silicon oxide [25].

According to the research results of silicon nanocrystals, because of similar atomic sizes of phosphorus atoms and silicon atoms, phosphorus doping will not cause significant changes in the crystal structure of silicon nanocrystals, whether it is distributed on the surface or enters the interior to form substitutional phosphorus [26]. As a result, there is no significant difference between the doped and undoped silicon nanosheets in the structural characterization of TEM (Figure 2d), HRTEM(inset of Figure 2d), XRD and Raman (not shown here).

**Figure 3:**
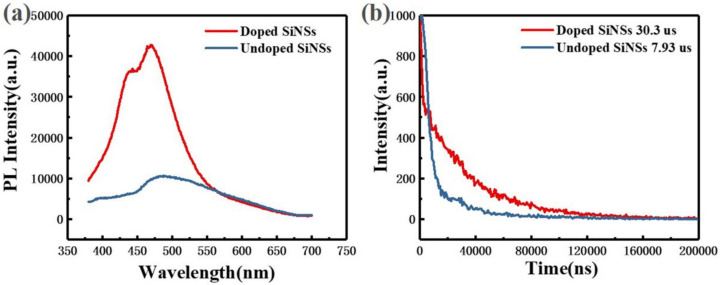
Optical characterization of undoped (blue line) and doped (red line) silicon nanosheets. (**a**) photoluminescence (PL) spectra; (**b**) transient photoluminescence (TR-PL) spectra.

Figure 3 shows the steady-state and transient photoluminescence (PL) spectra of undoped and doped silicon nanosheets. The luminescence peak of the undoped sample is approximately 480 nm. According to the theory of luminescence mechanism of silicon nanocrystals in the literature, the 480 nm luminescence peak may come from indirect band gap recombination of silicon or surface state recombination. According to the luminescence model of porous silicon proposed by Li et al. [27], luminescence of microsecond lifetime comes from the silicon particle core and nanosecond lifetime comes from the surface oxide layer. It can be seen from Figure 3b that the lifetime of the undoped sample conforms to the lifetime order of the indirect bandgap recombination. The PL spectrum of the doped sample also showed the luminescence peak at 480 nm derived from the same radiation recombination process. However, the luminescence intensity of the doped sample at this wavelength is approximately 4 fold that of the undoped sample. This is because the introduction of phosphorus impurities reduces the surface defect states and inhibits the non-radiative recombination process in silicon nanosheets. The surface passivation effect of phosphorus impurities is also reflected in the lifetime. The decay time of the doped sample at 480 nm (approximately 30.3 µs) is approximately 4 fold that of the undoped sample (approximately 7.93 µs).

In addition, a new luminescence peak appears in the doped sample at approximately 440 nm. By comparing the PL spectrum of the doped silicon nanosheets with the PL data in the literature [17,28], we believe that the luminescence peak is derived from the quasi-direct band gap transition of silicon nanosheets with thickness close to that of a single layer. Without doping treatment, these ultra-thin silicon nanosheets are unstable in the air and easily oxidized resulting in lack of luminescence of 440 nm.

**Figure 4:**
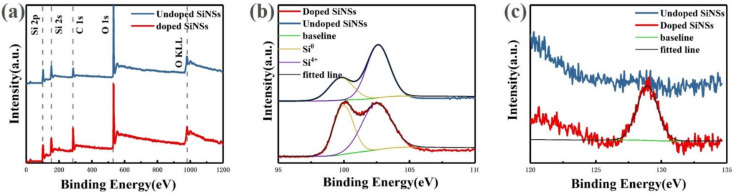
X-ray photoelectron spectroscopy (XPS) spectra of silicon nanosheets: (**a**) full spectra of undoped (blue line) and doped (red line) samples; (**b**) Si_2p_ spectra of undoped (blue line) and doped (red line) samples; (**c**) P_2p_ spectra of undoped (blue line) and doped (red line) samples.

Figure 4a shows the X-ray photoelectron spectroscopy (XPS) full spectra for both undoped and doped samples. It can be seen from the figure that the number and position of peaks in the two spectra are basically the same. The identification results of these peaks show that the main elements in the sample are silicon and oxygen, and no other elements remain. The semi-quantitative analysis shows that the Si/O ratio of the undoped sample is 0.67. The oxidation degree of the undoped sample is relatively serious, because there are a lot of dangling bonds on the surface of the silicon nanosheet, which has stronger surface reactivity than bulk silicon and is easier to be oxidized in the air. While the doped sample is less oxidized, with a Si/O ratio of 0.8. This is because part of the dangling bond is passivated by phosphorus atoms, which increases the stability of the silicon nanosheet in the air.

For undoped and doped samples, two Si_2p_ binding energy peaks located at 99 and 103 eV are detected which are corresponding to Si^0^ state and oxidized Si^4+^ state, respectively. By comparing the area of the two peaks, the content ratio Si^0^/Si^4+^ is approximately 0.4 in the undoped sample. For doped sample, this value is approximately 0.6. It can be seen from the ratios of these two state that although a certain degree of oxidation of silicon nanosheets is inevitable in the process of preparation, storage and testing, the degree of oxidation can be effectively reduced by passivation treatment.

Figure 4b shows the P_2p_ spectra of undoped and doped samples. No phosphorus-related signal was observed in the spectrum of undoped samples. The doped sample shows a peak at approximately 129 eV. According to the literature, the binding energy of the P-O bond and the P-Si bond is 134.5 and 128.4 eV, respectively [29], thus it is judged that the peak corresponds to P-Si bond. The appearance of the P-Si signal proves that phosphorus doping is achieved successfully by the spin-on-dopant method. According to the above test results of optical properties, it is inferred that phosphorus does not enter the inside of silicon nanosheets to form substitution doping but distributes on the surface. Therefore, the addition of phosphorus does not cause Auger recombination to reduce the luminescence performance of silicon nanosheets but enhances its luminescence by passivating the surface.

**Figure 5:**
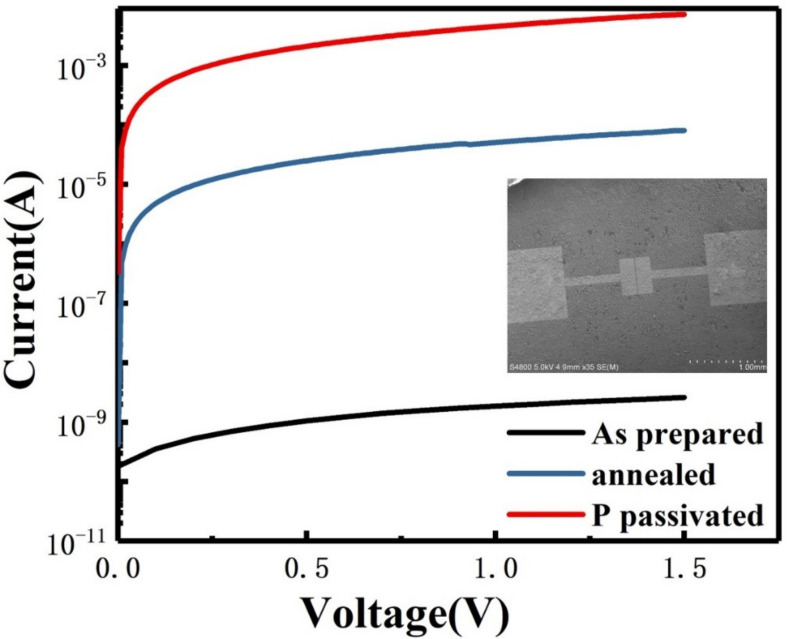
I–V characteristics of as-prepared (black line), annealed (blue line) and P passivated (red line) silicon nanosheets with SEM image of two-end device as inset.

The device prepared for electrical testing was shown as the inset in Figure 5. Silicon nanosheets suspension is dripped on SiO_2_/Si substrate, and 100 nm gold electrodes are evaporated on the sample, where the channel length is 25 µm and the width is 100 µm. By fitting the I–V curve, it can be found that the resistivity of the undoped silicon nanosheets is significantly higher than that of silicene in the literature [16,30]. This is because there are a lot of defects on the surface of silicon nanosheets synthesized by soft-chemical procedure, which become a trap of charge carriers. Moreover, in this device, the conduction process does not strictly occur within a single nanosheet. It also involves the carrier transmission between the interfaces of the nanosheets. The surface oxidation layer and interspace between adjacent nanosheets will impede the transmission and thus reduces the conductivity. Based on the above problems, we further improve the conductivity of multilayer silicon nanosheets by annealing at high temperature and phosphorous passivation. The edge contact parts of adjacent silicon nanosheets can be better connected or re-bonded by annealing. It can be seen from the blue line in Figure 5 that the resistance value of the silicon nanosheets after annealing is reduced by 4 fold compared with that of as-prepared samples. Due to the self-cleaning effect of nano-sized materials, the diffused phosphorus impurities are basically distributed on the surface of silicon nanosheets. Compared with silicon, the more electrons produced by phosphorus can neutralize the charged carrier trap on the surface. It can also be found that phosphorus passivation can further reduce the resistance value by 2 fold magnitude compared with that of annealing samples as shown by the red line in Figure 5.

Furthermore, the breakdown voltage of the doped sample is below 5 V and that of the undoped sample is above 20 V. For silicon, the critical value between tunnel breakdown and avalanche breakdown is approximately 5 V. We can infer that the breakdown mechanism of the undoped sample is avalanche breakdown under the high voltage. However, the increase in carrier concentration in the doped sample leads to the decrease in the width of barrier width and the increase in tunneling probability, so that the breakdown mechanism transforms into tunnel breakdown. According to the relationship curve between the breakdown voltage of bulk silicon and the impurity concentration, the doping concentration of silicon nanosheets is in the range of 10^18^ ~ 10^19^ cm^−3^.

Table 1 compares the resistivity of silicon nanosheets prepared by different methods. Due to the limitation of crystal quality and surface defects, the resistivity of silicon nanosheets prepared by soft chemical methods is much higher than that prepared by MBE and CVD, which has been fully explained in the introduction. Compared with other soft chemical prepared silicon nanosheets, our sample effectively reduces the resistivity, and the conductivity is closer to that of silicon nanosheets with high crystal quality. It is worth noting that the film thickness we used in the calculation of resistivity comes from the value of 4 µm measured in the SEM cross-section image (Figure 2b). It can be seen from the SEM cross-section image that there are a lot of gaps in the multilayer silicon nanosheets, so the actual thickness of the sample should be less than the measured value, and the actual resistivity of the sample may be lower.

## 4. Conclusions

A large number of ultra-thin silicon nanosheets were prepared by the de-intercalation reaction, and their quasi-two-dimensional properties were proved by the morphology and structure characterization. According to the results of EDS and XPS, the spin-on-dopant method successfully achieved phosphorus doping of silicon nanosheets prepared by the soft chemical method. The optical performance test shows that the luminescence peak of the undoped ultra-thin silicon nanosheet is 480 nm, which is derived from the indirect band gap recombination of silicon. By passivation of surface defects, phosphorus doped sample enhance the luminescence intensity to approximately 4 fold that of the undoped sample and extend the lifetime to approximately 4 fold. In addition, since phosphorus can achieve surface passivation and improve the stability of silicon nanosheets, phosphorus doped samples can also recover the quasi-direct band gap transition of silicon nanosheets at approximately 440 nm, which is easily quenched due to the existence of surface defects and surface oxidation. Electrical performance tests show that the surface defects can be passivated by phosphorus doping, which reduces the resistivity of multilayer silicon nanosheets by 6 fold. In conclusion, phosphorus doping can effectively overcome the adverse effects caused by a large number of surface defects of silicon nanosheets prepared by the soft chemical method and improve the optical and electrical properties of silicon nanosheets simultaneously.

## Figures and Tables

**Table 1:** Electrical resistivity of silicon nanosheets prepared by different methods.

Sample	Preparation Method	Resistivity (Ω · cm)	Reference
Silicene FETs	MBE	0.1–1.8^−2^	[16]
Si nanosheets(doped)	CVD	1.6 × 10^−2^	[31]
Pelletized nanosheets	Soft chemical method	7.25	[20]
Silicane FETs	Soft chemical method	1.89 × 10^4^	[32]
Si nanosheets(doped)	Soft chemical method	3.43 × 10^−1^	Present work

## Data Availability

All experimental data to support the findings of this study are available upon request by contacting the corresponding authors.
